# The TREATT Trial (TRial to EvaluAte Tranexamic acid therapy in Thrombocytopenia): safety and efficacy of tranexamic acid in patients with haematological malignancies with severe thrombocytopenia: study protocol for a double-blind randomised controlled trial

**DOI:** 10.1186/s13063-019-3663-2

**Published:** 2019-10-15

**Authors:** Lise J. Estcourt, Zoe McQuilten, Gillian Powter, Claire Dyer, Eleanor Curnow, Erica M. Wood, Simon J. Stanworth, on behalf of the TREATT Trial Collaboration (provisional)

**Affiliations:** 1 NHS Blood and Transplant, Oxford, UK; 2 Radcliffe Department of Medicine, University of Oxford, Oxford, UK; 3 Transfusion Research Unit, Department of Epidemiology and Preventive Medicine, Monash University, Melbourne, Australia; 4 Department of Haematology, Monash Health, Melbourne, Australia; 5 NHS Blood and Transplant Clinical Trials Unit, Headington, Oxford, UK; 6 NHS Blood and Transplant Statistics and Clinical Studies, Stoke Gifford, Bristol, UK

**Keywords:** Anti-fibrinolytic, Bleeding, Chemotherapy, Haematological malignancy, Haematopoietic stem cell transplant, Leukaemia, Lymphoma, Thrombocytopenia, Tranexamic acid

## Abstract

**Background:**

Patients with haematological malignancies often develop thrombocytopenia as a consequence of either their disease or its treatment. Platelet transfusions are commonly given to raise a low platelet count and reduce the risk of clinical bleeding (prophylaxis) or stop active bleeding (therapy). Recent studies have shown that many patients continue to experience bleeding despite the use of prophylactic platelet transfusions. Tranexamic acid is an anti-fibrinolytic, which reduces the breakdown of clots formed in response to bleeding. Anti-fibrinolytics have been shown to prevent bleeding, decrease blood loss and use of red cell transfusions in elective and emergency surgery, and are used widely in these settings. The aim of this trial is to test whether giving tranexamic acid to patients receiving treatment for haematological malignancies reduces the risk of bleeding or death and the need for platelet transfusions.

**Methods:**

This is a multinational randomised, double-blind, placebo-controlled, parallel, superiority trial. Patients will be randomly assigned to receive tranexamic acid (given intravenously or orally) or a matching placebo in a 1:1 ratio, stratified by site. Patients with haematological malignancies receiving intensive chemotherapy or stem cell transplantation (or both) who are at least 18 years of age and expected to become severely thrombocytopenic for at least 5 days will be eligible for this trial. The primary outcome of the trial is the proportion of patients who died or had bleeding of World Health Organization grade 2 or above during the first 30 days of the trial. We will measure the rates of bleeding daily by using a short, structured assessment of bleeding, and we will record the number of transfusions given to patients. We will assess the risk of arterial and venous thrombosis for 120 days from the start of trial treatment.

**Discussion:**

This trial will assess the safety and efficacy of using prophylactic tranexamic acid during a period of intensive chemotherapy and associated thrombocytopenia in people with haematological disorders.

**Trial registration:**

This study was prospectively registered on Current Controlled Trials on 25 March 2015 (ISRCTN73545489) and is also registered on ClinicalTrials.gov (NCT03136445).

**Electronic supplementary material:**

The online version of this article (10.1186/s13063-019-3663-2) contains supplementary material, which is available to authorized users.

## Background

Patients with haematological malignancies often develop severe thrombocytopenia, as a consequence of either their disease or its treatment, including chemotherapy and stem cell transplantation. Platelet transfusions are commonly administered, in this situation, to raise the low platelet count and reduce the risk of clinical bleeding (prophylaxis) or stop active bleeding (therapy). Many audits have indicated that the most common indication for administration of platelet transfusions to thrombocytopenic patients with haematological malignancies is prophylaxis (up to 69%) [1–7].

However, recent studies have raised questions about the effectiveness of platelet transfusions to reduce clinical bleeding. First, many patients experience bleeding despite the use of prophylactic platelets with World Health Organization (WHO) grade 2 or greater bleeding reported in 48% to 79% of patients receiving prophylactic platelets in clinical trials [8, 9]. Second, the absolute platelet count has not been shown to be strongly predictive of bleeding risk in this patient population [8].

Furthermore, platelet transfusions are not without risks and are a limited and expensive resource and therefore use should be targeted to those most likely to benefit. Adverse events may range from mild reactions, such as fever (one in five transfusions) [10], to more serious and even life-threatening events such as bacterial sepsis from transfusion transmitted infection (one in 10,000 transfusions) [10] or transfusion-related acute lung injury (TRALI) [11]. Patients may also become refractory to platelet transfusions, the incidence of which increases with the number of platelet transfusions a patient receives [12]. Once a patient becomes refractory, the ability to treat bleeding with platelet transfusions becomes more difficult, requiring expensive and specially matched platelet transfusions that are difficult to source. Demand for platelet components is rising in many countries, raising concerns about future shortages in the supply of platelets.

### Need for a trial

Studies have shown that a significant proportion of patients develop bleeding at some stage during the period of thrombocytopenia despite prophylactic platelet transfusions [8, 9]. There is also a lack of a relationship between the morning platelet count and bleeding risk, except at very low platelet counts. The PLADO (platelet dose) trial reported that patients had similar rates of bleeding (17%) with morning platelet counts within the wide range of 6 to 80 × 10^9^/L [8]. Platelet transfusion trials of dose and threshold, including the two largest studies [8, 13], have shown no difference in haemostatic outcomes between trial arms (i.e., no increased bleeding in the restrictive policy arms for transfusion by lower threshold or dose). The burden of bleeding varies in sub-groups of patients (e.g., higher bleeding rates in patients with acute leukaemia or receiving an allogeneic stem cell transplant (SCT) and lower rates in patients receiving an autologous SCT) [14]. These findings suggest that factors other than absolute platelet count may be important in prevention of bleeding in patients with haematological malignancies and that current policies for prophylactic platelet transfusions have a limited role in reducing much of the bleeding seen in haematology patients undergoing intensive chemotherapy or stem cell transplantation or both.

Any treatment that could reduce reliance on platelet transfusion support would have major cost-saving implications. Around 319,000 adult doses of platelets are issued in the UK each year [15] at an annual cost of about £64 million [16] and up to two thirds (67%) of these are given to patients with haematological malignancies [1, 4, 17].

There is a need for new treatment strategies to minimise the burden of bleeding, particularly in high-risk groups of patients. One approach is to administer anti-fibrinolytics. The fibrinolytic system acts to prevent blood clots from growing excessively away from the site of damage; conversely, delayed processes of breakdown of fibrin clots would be expected to enhance localised clot formation and, crucially, stability.

### Tranexamic acid

Tranexamic acid (TXA) is an anti-fibrinolytic drug – a lysine analogue that is a competitive inhibitor of plasminogen activation and, at higher concentrations, non-competitive inhibitor of plasmin [18]. TXA is the only lysine analogue currently licensed in the UK and Australia. It has been widely used in trauma, obstetrics and surgery to prevent [19] and reduce clinical bleeding and need for transfusion [20–22], but few studies have evaluated its use in patients with haematological malignancies [23]. A systematic review identified three small randomised controlled trials comparing TXA with placebo; all were published over 20 years ago and had significant methodological limitations. However, their results suggested a reduction in platelet transfusion usage when platelet transfusions were given only if patients bled [24, 25] and a possible reduction in the number of bleeds [24]. Two other ongoing studies are assessing prophylactic use of TXA in adults with haematological malignancies: A-TREAT was developed in conjunction with this trial and is being conducted in the US (NCT02578901), and PATH (NCT02650791) is comparing TXA with prophylactic platelet transfusions in adults undergoing an autologous SCT [26, 27].

### Study hypothesis

The study hypothesis is that, in patients with haematological malignancies, prophylactic use of anti-fibrinolytics during a period of severe thrombocytopenia will decrease bleeding and the demand for platelet transfusions. There may be additional benefits for patients, such as improved quality of life during their in-patient stay and earlier discharge home. Given the importance of understanding the cost-effectiveness of new treatments, this trial also incorporates a health economics analysis.

## Methods

### Study design and setting

This is a multinational, randomised, double-blind, placebo-controlled, parallel, superiority trial. Patients will be recruited in participating haematology departments in Australia and the UK. The current list of participating hospitals is available (https://clinicaltrials.gov/ct2/show/NCT03136445). Participants will be randomly assigned to receive TXA (given intravenously or orally) or a matching placebo in a 1:1 ratio, stratified by site. Randomisation will be further balanced within blocks of varying undisclosed sizes.

### Eligibility

People will be eligible for the trial if they are at least 18 years of age, have a confirmed diagnosis of a haematological malignancy, are being treated with chemotherapy or a haematopoietic SCT that is expected to lead to significant hypoproliferative thrombocytopenia (platelet count of ≤10 × 10^9^/L for ≥5 days), and are able to comply with treatment and monitoring.

People will be excluded from the trial if they have a history or current diagnosis of arterial or venous thromboembolic disease, including myocardial infarction, peripheral vascular disease and retinal arterial or venous thrombosis; a diagnosis of acute promyelocytic leukaemia and undergoing induction chemotherapy; a diagnosis/history of veno-occlusive disease (VOD) (also called sinusoidal obstruction syndrome); known inherited or acquired prothrombotic disorders; history of immune thrombocytopenia, thrombotic thrombocytopenic purpura or haemolytic uraemic syndrome; overt disseminated intravascular coagulation; required a platelet transfusion threshold of more than 10 × 10^9^/L at time of randomisation (this excludes a transient rise in the threshold due to sepsis); known inherited or acquired bleeding disorder, such as acquired storage pool deficiency; paraproteinaemia with platelet inhibition; receiving anti-coagulant therapy or anti-platelet therapy; visible haematuria at time of randomisation; anuria (defined as urine output of less than 10 mL/h over 24 h); severe renal impairment (estimated glomerular filtration rate of not more than 30 mL/min per 1.73 m^2^); history of epilepsy, convulsions, fits or seizures; pregnant or breast-feeding; allergic to TXA; enrolled in other trials involving platelet transfusions, anti-fibrinolytics, platelet growth factors or other pro-coagulant agents; or were previously randomly assigned into this trial at any stage of their treatment.

### Intervention

Trial treatment will be started as per randomisation assignment as soon as possible within 24 h, and no later than 72 h, of the first recorded platelet count of not more than 30 × 10^9^/L or if the participant was admitted with a platelet count already below 30 × 10^9^/L as soon as possible within 24 h, and no later than 72 h, after the start of chemotherapy or conditioning for an SCT. It will be continued until the platelet count is more than 30 × 10^9^/L for three consecutive days without platelet transfusion support or until the participant has received 30 days of treatment.

The trial treatment (TXA 1 g or placebo) will be administered intravenously as a slow intravenous bolus or infusion over 10 min every 8 h. If the participant is well enough, they can switch to oral trial treatment (TXA 1.5 g/placebo) three tablets every 8 h.

For patients with mild to moderate renal impairment, the dosage of trial treatment will be reduced according to the serum creatinine level (Tables 1, 2 and 3).

**Table 1:** Dose adjustment for renal impairment

Serum creatinine		Dose IV	Dose PO	Oral suggested dose		Administration
μmol/L	mg/10 mL					
120 to 249	1.35 to 2.82	10 mg/kg BW	15 mg/kg BW	<50 kg	500 mg	Every 12 h
				50 to 83 kg	1 g	
				84 to 116 kg	1.5 g	
				>116 kg	2 g	
250 to 500	2.82 to 5.65	10 mg/kg BW	15 mg/kg BW	<50 kg	500 mg	Every 24 h
				50 to 83 kg	1 g	
				84 to 116 kg	1.5 g	
				>116 kg	2 g	
>500	>5.65	5 mg/kg BW	Omit dose			Every 24 h

*Abbreviations*: *BW* body weight, *IV* intravenous, *PO* by mouth

**Table 2:** Trial schedule

Trial assessment	Enrolment (consent)	Days between enrolment and randomisation	Day R Day of randomisation platelet count ≤50 × 10^9^/L	Days between day R and study day 1	Day 1	Day 2	Days 3–11	Day 12 (± 2)	Days 13–29	Day 30 (± 2)	Day 60 (± 3)	Day 120 (± 14)
Demographics and medical history	X											
Eligibility assessment	X		X		X (Prior to starting drug)							
Informed consent	X											
Transfusion requirements			X	X	X	X	X	X	X	X		
Bleeding assessment			X	X	X	X	X	X	X	X		
Trial treatment accountability					X	X	X	X	X	X		
Quality-of-life assessment			X					X		X		X
Health economic evaluation										X		X
Thrombotic assessment	Medical notes		Medical notes	Medical notes	Medical notes	Medical notes	Medical notes	Medical notes	Medical notes	Face-to-face or telephone follow-up		Face-to-face or telephone follow-up
Highest recorded temperature each day			X		X	X	X	X	X			
Serious adverse event assessment			X	X	X	X	X	X	X	X	X

**Table 3:** Trial schedule (investigations)

Trial assessment	Enrolment	Days between enrolment and randomisation	Day R Day of randomisation platelet count ≤50 × 10^9^/L	Days between Day R and study day 1	Day 1	Day 2	Days 3–11	Day 12 (± 2)	Days 13–29	Day 30 (± 2)
Pregnancy test (if applicable)	X									
Urine dipstick	X									
Haemoglobin	X		X		X	X	X	X	X	X
Platelet count	X	X	X	X	X	X	X	X	X	X
Prothrombin time (or INR if PT not available)	X		X							
Serum creatinine (U&E)	X		X		X	X	Three times a week or as SOC	X	Three times a week or as SOC	X
Liver function tests: bilirubin and albumin	X		X				Required if VOD is reported/suspected 3 times a week		Required if VOD is reported/suspected 3 times a week	
HLA antibody screen†	X									
INVESTIGATIONS TO BE PERFORMED ONLY AT SELECTED PARTICIPATING CENTRES										
Assays for fibrinolysis	X		X			X			Required three times a week	

*Abbreviations*: *INR* international normalised ratio, *PT* prothrombin time, *SOC* standard of care, *U&E* urea and electrolytes, *VOD* veno-occlusive disease, *X* measurement required

† HLA antibodies to be rechecked if participant becomes refractory to platelet transfusions. Please see section 6.4.1

The trial treatment will be permanently discontinued as soon as any one of the following situations occurs: it has been 30 days since the trial treatment has started; the participant has a spontaneous increase in platelet count from less than 30 × 10^9^/L to more than 50 × 10^9^/L or has had three consecutive days with morning platelet counts of more than 30 × 10^9^/L and no requirement for platelet or granulocyte transfusion or SCT; the participant is treated with open-label TXA, other anti-fibrinolytic agent or pro-coagulant drug, anti-coagulant or anti-platelet drug; the participant has visible haematuria; the participant has a diagnosis of thrombosis; the participant becomes anuric (defined as urine output of less than 10 mL/h over 24 h); or the participant develops sinusoidal obstructive syndrome (SOS) (also called VOD). In addition to the reasons stated above, participants may stop treatment early or be stopped early for any of the following reasons: haematological disease progression, unacceptable adverse reaction to trial treatment, any change in the participant’s condition that justifies the discontinuation of treatment in the opinion of the clinician, or withdrawal of consent.

Daily trial treatment accountability will be performed. Any unused medication will be collected by the research nurse from the ward (if participant is an in-patient) or participant (if participant is an out-patient) and returned to the local trial pharmacist. The pharmacist will document the amount dispensed and returned for each study participant.

### Concomitant care

Patients will receive platelet and red cell transfusions in accordance with national guidelines. Prophylactic platelet transfusions will be given at threshold counts of less than or equal to 10 × 10^9^/L. Therapeutic platelet transfusions may also be given following objective and documented signs or symptoms of bleeding at WHO grade 2, 3 or 4 or in accordance with the local physicians’ usual practice. Prior to planned invasive procedures, physicians will be allowed to increase the transfusion dose or threshold (or both) in keeping with their current practice. Clinicians can exercise discretion to transfuse platelets for any reason should they feel there is a clinical reason to do so; the rationale must be clearly recorded on the daily transfusion data form.

If a patient develops platelet refractoriness (defined as two sequential transfusions with a 24-h platelet increment of less than 5 × 10^9^/L), a serum sample will be drawn for lymphocytotoxic antibodies. If the panel reactive antibody (PRA) is at least 20%, the patient will be presumed to be alloimmune platelet refractory and may be given either HLA-matched or cross-match compatible platelet transfusions. If the PRA is less than 20%, local practice will be followed to treat the refractoriness. The patient will remain in the trial and data will continue to be collected on the patient.

### Primary outcome

The primary outcome is the estimated proportion of participants who died or had bleeding of WHO grade 2 or above during the first 30 days of the trial. Day 1 is the first day of trial treatment.

### Secondary outcomes

Secondary efficacy outcomes are all measured during first 30 days of the trial. These include the proportion of days with bleeding (WHO grade 2 or above), time to first episode of bleeding of WHO grade 2 or greater for those participants who bled, highest grade of bleeding a participant experiences, number of platelet transfusions per participant, number of red cell transfusions per participant, proportion of participants surviving at least 30 days without a platelet transfusion, proportion of participants surviving at least 30 days without a red cell transfusion, and quality of life.

Secondary safety outcomes include the number of thrombotic events from first administration of trial treatment up to and including 120 days after the first dose of trial treatment is administered, per day at risk; number of participants developing VOD (SOS) within 60 days of first administration of trial treatment; all-cause mortality during the first 30 days and the first 120 days after the first dose of trial treatment is administered; death due to thrombosis during the first 120 days after the first dose of trial treatment is administered; death due to bleeding during the first 30 days after the first dose of trial treatment is administered; and number of serious adverse events from first administration of trial treatment until 60 days after the first dose of trial treatment is administered.

### Exploratory outcomes

These will all be measured during the first 30 days of the trial. They include the proportion of days with thrombocytopenia; proportion of days with fever (highest daily temperature of at least 38.1 °C) of days spent in hospital; reasons for platelet and red cell transfusions; trial treatment summary (changed route or frequency, missed dose, reasons for discontinuation of treatment, and number of days on trial treatment and on oral trial treatment); number of granulocyte transfusions per participant; location of any bleeding; number of participants requiring interventions and procedures for bleeding; number of participants requiring concomitant medication; and number of participants on gemtuzumab ozogamicin.

### Sub-group analyses

Sub-group analyses will be performed for the primary outcome for the following variables in the main analysis:

- Country of participant (UK versus Australia), if heterogeneity between UK and Australian participants is identified in the interim analysis
- Platelet count at consent (≤30 × 10^9^/L versus >30 × 10^9^/L)
- Treatment compliance during the first 30 days of the trial

### Recruitment and consent

Centre selection will be based on the presence of appropriate clinical and research infrastructure, including adequate local resources and facilities to support recruitment, and adequate qualified staff to conduct the trial properly and safely.

**Fig. 1:**
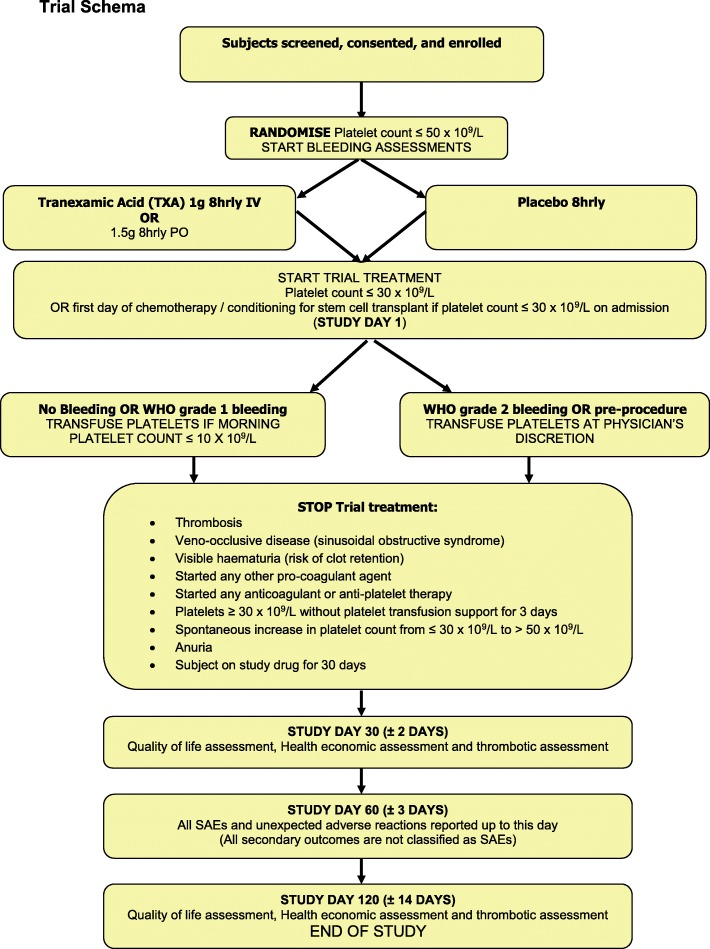
Trial schema

Patients will be approached within a hospital setting and screened for eligibility by research staff to ensure that all inclusion and exclusion criteria are met (Fig. 1). Informed consent to enter the trial is obtained from a patient by a clinician only after a full explanation has been given, a patient information sheet has been provided, and time has been allowed for consideration (Additional file 1).

### Sample size

Based on the experience of similar patients in the TOPPS trial [9], in the absence of anti-fibrinolytic therapy, it is anticipated that 43% of eligible patients will experience death or WHO grade 2 bleeding or higher within the first 30 days of the TREATT trial. We judged that a 26% relative reduction in bleeding rates would be sufficient to change clinical practice because it would be necessary to treat only 8.9 patients to have an impact on 1 patient provided that no new safety issues related to anti-fibrinolysis in the thrombocytopenic population are uncovered. We thus evaluated the planned sample size of the trial relative to this hypothesised effect. We plan to recruit 616 participants to the TREATT trial. The primary endpoint will be analysed by using the Kaplan–Meier (KM) method to estimate the probability of bleeding or death within 30 days in the analysis populations. Power and sample size calculations are based on a log-rank test for comparing two survival curves, as described in Collett [28]. Under the assumption that anti-fibrinolysis results in a decrease of death or WHO grade 2 bleeding from 43% to 32%, the 616 patients planned to be recruited will provide 80% power to detect an observed absolute decrease in bleeding rates of 11% (a relative reduction of 26% with a number needed to treat (NNT) of 8.9) and will be judged statistically significant at the two-sided 0.05 level. The planned trial size of 616 subjects includes an additional 5% of patients above the required sample size of 586. This 5% figure is more than the proportion of patients within TOPPS (4%) who dropped their platelet count below 50 × 10^9^/L but did not drop their platelet below 30 × 10^9^/L. This therefore accounts for the number of patients within the trial who are randomly assigned but never receive the trial treatment.

We do not expect there to be a problem with the assessment of the primary outcome since, in the recent TOPPS trial conducted by our group and using the same bleeding assessment tool, completeness of bleeding outcome documentation was excellent. A bleeding assessment was completed on 95% (17,138/18,000) of study days. The majority of patients had bleeding information completed on each trial day (median of 30 days). There were also only 6 deaths (1% of patients) while on the trial.

This trial is not powered to definitively establish the safety of the treatment with respect to the frequency of venous thromboembolism (VTE). However, with the planned enrolment of 616 patients, observed differences in frequency of VTE of 3.6% in the placebo arm and 5% or less in the TXA arm would result in a 95% confidence interval that excluded a relative risk of 3.5.

### Randomisation

Participants will be randomly assigned to receive TXA or a matching placebo in a 1:1 ratio, stratified by site, using an online randomisation service (SealedEnvelope). Randomisation will be balanced within blocks of varying undisclosed sizes. An independent unblinded statistician will generate the randomisation list. A site-specific list of coded treatment allocations will also be provided to the trial pharmacist at each site by the same independent statistician to enable the pharmacist to dispense the correct trial treatment. Supplies of active and placebo trial treatment will be provided to the hospital pharmacies and labelled with a code so the pharmacist does not know which is active and which is placebo.

### Blinding and unblinding

In this trial, the participants, the principal investigator (PI), the ward nurses, ward pharmacist, all other site staff, and all members of the trial management group (TMG) will be blinded to treatment allocation. The clinical trial pharmacists at each centre will be semi-blinded in that they will know which participants are in each group but not what the group is.

In general, there should be no need to unblind the allocated treatment. Unblinding will be carried out only in those rare cases when the clinician believes that clinical management depends importantly upon knowledge of whether the patient is receiving TXA or placebo. In those few cases when urgent unblinding is considered necessary, the PI will be given access to the web-based service (www.sealedenvelope.com).

### Bleeding assessments

Daily data collection and bleeding assessment will commence when the patient is randomly assigned and will stop when one of the following occurs:

- It has been 30 days since the first recorded platelet count was not more than 30 × 10^9^/L.
- The participant dies.
- The participant withdraws his or her consent to having bleeding assessments performed.
- The site investigator withdraws the participant from all further study assessments.

In the TREATT trial, a number of measures will be taken to standardise documentation and recording of bleeding, including trained assessors, monitoring and education. Bleeding assessments will be conducted by using a tool based upon that used in the recent TOPPS trial [9] and further developed and validated by an international working group: the BEST collaborative [29, 30].

In the TOPPS trial [9], in which completeness of bleeding outcome documentation was excellent, a bleeding assessment was completed on 93% (8405/9030) of days for patients in the no-prophylaxis group and 97% (8733/8970) of days in the prophylaxis group. The majority of patients in both arms had bleeding information collected on each trial day (median no-prophylaxis 30 days (interquartile range (IQR) 29 to 30) and median prophylaxis 30 days (IQR 30 to 30)).

For in-patients, bleeding assessments will be performed by using the bleeding assessment tool which includes a physical assessment of the patient, patient interview (if possible) and review of patient chart and laboratory data. Research staff will perform the physical assessment and interview before reviewing the patient’s charts, medical notes and laboratory data to allow an objective assessment. Research staff will perform the bleeding assessment at approximately the same time each day.

All patients who are discharged home before completion of the trial study period (30 days from platelet count falling to not more than 30 × 10^9^/L) will be asked to complete a daily diary. They will be asked to respond with a yes/no answer as to whether they have experienced any bleeding on that day. If the answer is yes, they will be instructed to contact their local haematology team for advice. If the local haematology team consider that the bleeding is clinically significant, it is likely that the patient will be asked to attend the out-patient clinic where a member of the local research team can perform a clinical bleeding assessment.

Additionally, all patients at home will be contacted by the local research staff on day 30 from first dose of the trial treatment to review the diary and confirm arrangements to collect all completed forms which will be forwarded to data management for entry into the trial database.

Local research staff completing the daily assessments will receive training and have guide notes to help them collect these data. Grade of bleed (WHO grading system) will be assigned centrally by means of a computer algorithm at the time of analysis.

### Safety assessments

Safety outcomes will include recording symptomatic thrombotic events that occur during the study period and up to 120 days (+/− 7) after the first dose of the trial treatment is administered. For in-patients, medical chart notes and imaging studies will be reviewed daily for documentation of any thrombotic events. For out-patients, the local research nurses will attempt to contact the participant directly; if contact is not made, the participants’ local physician/general practitioner (GP) will be contacted.

**Fig. 2:**
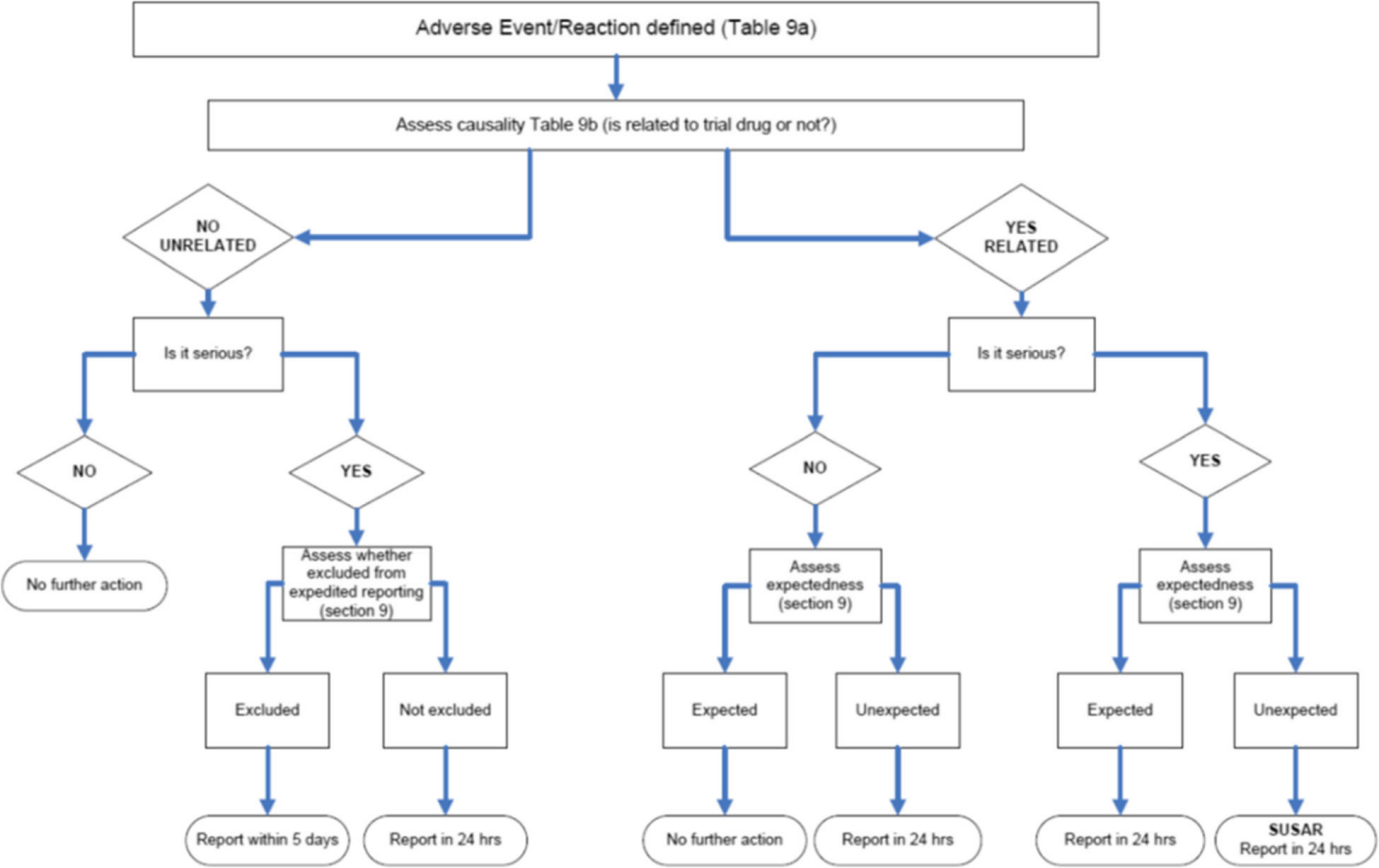
Assessment of adverse events/reactions

Local investigators will assess the causality and seriousness of adverse events and report all unexpected adverse reactions or serious adverse events until day 60 of the trial (Fig. 2). After discharge from hospital, a member of the research team will contact the participant in person or by telephone to ask them about any interval medical events or serious adverse events. If the research team are unable to contact the patient directly, their local physician/GP will be contacted.

### Health economics analysis

A cost-utility analysis will be conducted for the main analysis; outcomes will be measured as quality-adjusted life years (QALYs) and calculated by combining data on patient survival with data on patient quality of life measured by using the EuroQol EQ-5D-5 L questionnaire. A secondary analysis will also consider a cost per bleed averted endpoint.

We will include data for the index admission on TXA, transfusions, treatment for any major bleeds, type and number of thromboembolic events, time in intensive treatment unit (ITU), time on ventilation, and time on general hospital ward. Post-discharge out to 120 days post-start of study drug, we will include data on readmissions to hospital and other hospital visits (out-patient clinic and day hospital attendances and attendances-and-emergency visits).

### Loss to follow-up

Consent will be obtained on enrolment to the trial to contact their family physician/GP if the local research team are unable to contact a participant to obtain follow-up data. Data will be recorded on all-cause mortality, mortality due to bleeding and mortality due to thrombosis.

Participants are free to stop the trial treatment at any time. The date will be recorded, along with the reason, on a designated trial treatment discontinuation form. In the event the patient stops the trial treatment, the patient will still be followed for all trial outcomes according to the protocol unless they have specifically withdrawn consent for further data collection. The trial end date for each participant will be the date of the last telephone call at study day 120.

### Data management

The NHS Blood and Transplant (NHSBT) Clinical Trial Unit (CTU) data managers will review all data received for errors and missing data points. Central monitoring procedures for data verification and issue and resolution of queries raised will be detailed in a separate data management plan.

The frequency, type and intensity for routine on-site monitoring and the requirements for “for cause” monitoring will be detailed in a separate monitoring plan.

Participating investigators agree to allow trial-related monitoring, including audits, ethics committee review and regulatory inspections by providing direct access to source data and documents as required; this is also included as part of patient consent. The data will be handled in accordance with the principles of the UK Data Protection Act.

### Statistical methods

All analyses will be adjusted for the stratification variable (recruitment site) by including a random site effect in each model.

### Efficacy population

The population used for efficacy analyses will be a modified intent-to-treat population including all eligible randomly assigned patients whose platelet count falls to 30 × 10^9^/L or below. Owing to the emergent nature of treatment of thrombocytopenic patients, participants are randomly assigned in a double-blind fashion to the treatment arms when platelet levels drop below 50 × 10^9^/L. This will allow time for the pharmacy to prepare the trial treatment and for the trial treatment to be available when the platelet count falls to 30 × 10^9^/L or below. This has been instituted for pragmatic reasons and will guard against delays in the availability of the trial treatment.

### Safety population

The population used for safety analyses will be all participants who receive any amount of trial treatment, using data gathered from first administration of trial treatment until up to 120 days after first administration of trial treatment.

### Primary efficacy analyses

1. The proportion of participants who die or have bleeding of grade 2 or above by WHO criteria during the first 30 days from the first dose of trial treatment, or planned first dose for those participants who did not receive treatment, will be estimated by the KM method and compared by using Cox regression analysis. All patients whose platelet counts dropped to 30 × 10^9^/L or below, regardless of length of follow-up, will be included.

### Secondary efficacy analyses

1. Proportion of days with bleeding up to study day 30; this will be compared by using logistic regression. Nesting at participant level will be accounted for by including a random participant effect.
2. Time to first episode of bleeding of WHO grade 2 or greater up to study day 30 will be estimated by using the cumulative incidence function, with the competing risk of death prior to bleeding of WHO grade 2 or greater. Cox regression analysis will be used to compare the two treatment groups. Participants who have not experienced bleeding of WHO grade 2 or greater will be censored at day 30 or at the point of last contact, whichever is first. Participants who died prior to study day 30 and did not experience bleeding of WHO grade 2 or greater will be censored at the time of death.
3. Highest grade of bleeding a participant experiences up to study day 30; this will be modelled by using an ordinal logistic regression model, modelling grade as an ordinal categorical outcome.
4. Number of platelet transfusions per participant up to study day 30 will be compared by using a negative binomial model. The model will include an offset to account for the number of days the participant spent in hospital (up to 30 days).
5. Number of red cell transfusions per participant up to study day 30 will be compared by using a negative binomial model. The model will include an offset to account for the number of days the participant spent in hospital (up to 30 days).
6. Proportion of participants surviving up to 30 days without a platelet transfusion will be estimated by the KM method and compared by using Cox regression analysis. The event of interest is time to first platelet transfusion or death.
7. Proportion of participants surviving up to 30 days without a red cell transfusion will be estimated by the KM method and compared by using Cox regression analysis. The event of interest is time to first red cell transfusion or death.
8. Quality of life at study days 12 and 30 will be assessed by using FACT-G and FACT-Th health questionnaires. For the FACT-G subscale scores (physical well-being, social/family well-being, emotional well-being, functional well-being), FACT-G total score and FACT-Th total score, mean (standard deviation) will be calculated and compared by treatment arm by using normal regression analysis.

### Secondary safety analyses

1. Number of thrombotic events from first administration of trial treatment up to and including 120 days after the first dose of trial treatment is received, per day at risk will be described by arm.
2. Number of participants developing VOD/SOS within 60 days of first administration of trial treatment will be described by arm.
3. All-cause mortality during the first 30 days and 120 days after the first dose of trial treatment is administered will be estimated by the KM method and compared by using Cox regression analysis.
4. Death due to thrombosis during the first 120 days after the first dose of trial treatment is administered will be estimated by using the cumulative incidence function, with the competing risk of death due to other causes. Cox regression analysis will be used to compare death due to thrombosis between treatment arms. Participants who have not died will be censored at day 120 or at the point of last contact, whichever is first. Participants who died prior to day 120 from causes other than thrombosis will be censored at the time of death. If numbers of deaths due to thrombosis are very small, the number of deaths will simply be described by arm.
5. Death due to bleeding during the first 30 days after the first administration of trial treatment is received will be estimated by using the cumulative incidence function, with the competing risk of death due to other causes. Cox regression analysis will be used to compare death due to bleeding between treatment arms.
6. Number of serious adverse events from first administration of trial treatment until 60 days after the first dose of trial treatment is administered, per day at risk, will be summarised by arm, including number of symptomatic thrombotic events (venous thromboembolisms and arterial ischaemic events), VOD, sepsis, organ failure and death.

### Other analyses

1. Proportion of days with thrombocytopenia (<10 × 10^9^/L, <30 × 10^9^/L, <50 × 10^9^/L) will be analysed by using logistic regression. Correlation at participant level will be accounted for by including a random participant effect. If the number of thrombotic events is very small, the proportions will simply be described by arm.
2. Proportion of days with fever (highest daily temperature ≥38.1 °C) of days spent in hospital, up to study day 30, will be analysed by using logistic regression.
3. Reasons for platelet and red cell transfusions; the reasons will be described by arm.
4. Trial treatment will be summarised by arm, including number of participants who changed route or frequency, number of participants who missed a dose, reasons for discontinuation of treatment, and median (IQR) number of days on trial treatment and on oral trial treatment.
5. Number of granulocyte transfusions per participant up to study day 30 will be summarised by treatment arm.
6. Location of bleeding up to study day 30 will be summarised by treatment arm.
7. Number of participants requiring interventions and procedures for bleeding up to study day 30 will be summarised by treatment arm.
8. Number of participants requiring concomitant medication (categorised as medication increasing risk of bleeding, medication decreasing risk of bleeding, and no concomitant medication) up to study day 30 will be summarised by treatment arm.
9. Number of participants on gemtuzumab ozogamicin during the first 30 days of the trial will be summarised by treatment arm.

### Trial monitoring

To provide protection for study participants, an independent data monitoring committee (DMC) has been appointed for this trial to oversee the safety monitoring. The DMC will review, on a regular basis, accumulating data from the ongoing trial and advise the trial steering committee (TSC) regarding the continuing safety of current participants and those yet to be recruited as well as reviewing the validity and scientific merit of the trial.

The DMC will be guided by a DMC charter that delineates the roles and responsibilities of the DMC, including delineation of the lines of communication between trial clinical investigators, trial data coordinating centre, and NHSBT.

In addition to potential audits by the Medicines and Healthcare products Regulatory Agency (MHRA) or the local research-and-development department, NHSBT reserves the right to conduct site audits either as part of its ongoing audit programme or in response to adverse observations during monitoring visits.

### Modifications of the protocol

Any modifications to the protocol that may impact on the conduct of the study, the potential benefits to the patients, or patient safety, including changes of study objectives, study design, population, sample sizes, study procedures, or significant administrative aspects, will require a formal amendment to the protocol. Such amendments will be reviewed and agreed by the TMG, the TSC and NHSBT as the sponsor prior to approval by the ethics committee and regulatory authorities.

### Trial status

This trial opened for recruitment on 23 June 2015 and is expected to complete recruitment by 31 March 2020. The current protocol version is 3.1 (dated 30 July 2018) and is available on request from the trial manager: gillian.powter@nhsbt.nhs.uk.

### Dissemination

The results from different centres will be analysed together and published as soon as possible. Individual clinicians must not publish data concerning their patients that are directly relevant to questions posed by the trial until the TMG has published its report. The TMG will form the basis of the writing committee and will advise on the nature of publications. Authorship of all publications associated with this trial will be compliant with the International Committee of Medical Journal Editors criteria for authorship. The TSC will see and approve the final trial publication. The CIs will see and approve any supplementary publications (e.g., from a sub-study). A trial identifier will be included on all presentations and publications (e.g., the ISCRTN).

## Additional files

Additional file 1: (DOCX 93 kb)

Additional file 2: SPIRIT (Standard Protocol Items: Recommendations for Interventional Trials) 2013 Checklist: Recommended items to address in a clinical trial protocol and related documents. (DOCX 37 kb)

## Data Availability

Individual participant data that underlie all published trial results will be available upon request from the NHSBT CTU after de-identification (text, tables, figures and Additional files). Beginning 9 months and ending 5 years following article publication, data will be shared with investigators whose use of the data has been assessed and approved by an NHSBT review committee as a methodologically sound proposal. Data will be shared to achieve the aims in the approved proposal.

## Abbreviations

DMC: Data monitoring committee
GP: General practitioner
IQR: Interquartile range
KM: Kaplan–Meier
NHSBT: NHS Blood and Transplant
PI: Principal investigator
PRA: Panel reactive antibody
SCT: Stem cell transplant
SOS: Sinusoidal obstructive syndrome
TMG: Trial management group
TSC: Trial steering committee
TXA: Tranexamic acid
VOD: Veno-occlusive disease
VTE: Venous thromboembolism
WHO: World Health Organization
