# Supplementary material for: The TREATT Trial (TRial to EvaluAte Tranexamic acid therapy in Thrombocytopenia): safety and efficacy of tranexamic acid in patients with haematological malignancies with severe thrombocytopenia: study protocol for a double-blind randomised controlled trial
Source: Trials. 2019 Oct 15;20:592. doi: 10.1186/s13063-019-3663-2 (PMC6792262; doi:10.1186/s13063-019-3663-2)
Supplement: Supplementary file 1 — (DOCX 93 kb) [file 13063_2019_3663_MOESM1_ESM.docx]

# Appendices

Appendix 1: Model Consent Form and patient information leaflet


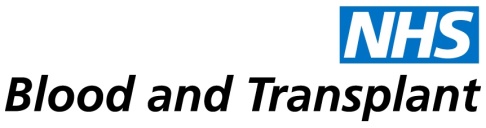
***Insert Hospital header***

**TREATT: Trial to Evaluate Antifibrinolytic Therapy in Thrombocytopenia**

Name of Investigator:_________________________ Participants Trial Number_______________

**CONSENT FORM**

Please initial all boxes

1. I confirm that I have read and understand the information sheet *(Insert version number and date)*  for the above study. I have had the opportunity to consider the information, ask questions and have had these answered satisfactorily.
2. I understand that my participation is voluntary and that I am free to withdraw at any time without giving any reason, without my medical care or legal rights being affected.
3. I understand that relevant sections of my medical notes and data collected during the study, may be looked at by the study investigators, regulatory authorities or from the NHS organisation, where it is relevant to my taking part in this research. I give permission for these individuals to have access to my records.
4. I understand that all information will be anonymised by the allocation of codes and that information will remain confidential and only be used for research
5. I agree to my GP being informed of my participation in the study, and to being approached for information relevant to the trial if necessary.
6. I agree to take part in the above study.

**For centres participating in fibrinolysis sub-study – optional consent**

- 1. I confirm that I have read and understood the information *(Insert version number and date)* for the fibrinolysis sub-study. I understand that blood samples taken for the study will be stored in a secure freezer and only identified by a code number, and that these samples may need to be sent outside the hospital for testing.
  2. I agree that any stored blood samples that remain after completion of this study may be used in future related studies that have been ethically approved.
  3. I agree to take part in the fibrinolysis sub-study.

Name of Participant Date Signature

Name of Person taking consent Date Signature

***Insert Hospital header***


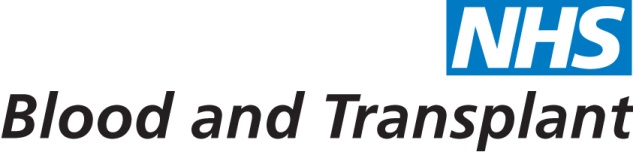


**PARTICIPANT INFORMATION SHEET**

**TREATT: TRial to EvaluAte Tranexamic acid therapy in Thrombocytopenia**

***Effectiveness of a supplementary drug treatment.***

REC reference number: 14/SC/1290

We are inviting you to take part in a research study. Before you decide whether to take part it is important for you to understand why the study is being done and what it will involve. Please take time to read the following information carefully and discuss it with friends and relatives if you wish. Ask us if there is anything that is not clear or if you would like more information.

**Why have I been invited?**

Patients with haematological diseases often develop a very low platelet count. This may be either as a result of the disease itself or the treatment, including chemotherapy and stem cell transplantation. It is expected that during your hospital stay you will develop a low platelet count. Patients with low platelet counts sometimes experience bleeding. Usually this is minor, for example some bruising or red blotches on your skin, or maybe a short nose-bleed. A platelet transfusion is often given to raise the low platelet count. But we now know that despite platelet transfusions, many patients continue to experience some bleeding when platelet counts are low.

What is the purpose of the study?

This study is to find out if giving a drug called tranexamic acid (TXA) whilst platelet counts are low will reduce bleeding. We would give this drug to some patients in addition to platelet transfusions.

**Do I have to take part?**

No, it is up to you to decide whether or not to take part. This choice will not affect your current or future care; your doctors and nurses will still care for you and give you all the treatments you need. You would be free to come out of the study at any time and without giving a reason. This will not affect the standard of care you receive.

How does the study work?

We don’t know whether TXA as well as all the usual treatments will be better at reducing bleeding or not. The best way to find out is to see how people who are given it do, compared to people who are not. This means that patients taking part in the study will be divided into two groups. Patients in one group will receive the study drug (TXA); patients in the other group will receive a placebo drug. The two groups will be similar so that a fair comparison can be made. The groups will be selected at random using a computer, which needs no personal information about you. The study drug and the placebo drug look the same so you, the doctors and the ward nurses don’t know which group you are in.

What will happen to me if I take part?

If you agree to take part, you will first be asked to sign a consent form. The research nurse will register you in the trial and check your daily blood results to see what your platelet count is. When your platelet count is 50 (50 x 10^9^/L) or less the trial computer system will allocate you to a treatment (study drug or placebo). Neither the research nurse nor the ward nurses will know what treatment you are having. When your platelet count is 30 (30 x 10^9^/L) or less the ward nurses will start the study treatment. To begin with you will receive the study treatment through a drip, 3 times a day. You can change to tablets if you are well enough to swallow them. There will be 3 tablets 3 times a day. You will continue with all your normal treatment as well.

Your doctor will arrange for you to have a platelet transfusion if your platelet count is very low, below 10 (10 x 10^9^/L), or if you have any signs of bleeding.

A member of the research team will check your ward charts, medical notes and blood results on a daily basis to collect blood results that we need for this study.

Participant’s responsibilities.

What do I have to do?

It is likely that you will be in hospital for some of your treatment. We would like to record signs and symptoms of bleeding when your platelet count is low. We know from experience that on many of these days you will not have any bleeding.

When you are on the ward, one of the research nurses helping with this study will come and see you each morning to ask you questions about signs and symptoms of bleeding and to look at your skin and in your mouth. This information will be recorded in a bleeding assessment form.

We would like to collect information about bleeding for 30 days after you start the study treatment. If you go home before the 30 days are finished, we will give you a simple diary card to fill in yourself with details of any signs or symptoms of bleeding.

If your platelet count is still low, less than 30 (*30 x 10^9^/L*), when you go home we will give you the study treatment to take as tablets by mouth 3 times a day until your platelet count is 30 or more or until you have been receiving the study treatment for 30 days.

We would like to ask you to complete 2 short questionnaires which have questions about your general wellbeing and quality of life. These would be completed on 2 occasions when you are in hospital. We would also like to contact you 1 month after starting the study treatment and again 3 months after that to see how you are. This contact could be either in the outpatient clinic or over the telephone.

What are the possible benefits of taking part?

We do not know if being in this study will help you, but it will help doctors who treat haematology patients in the future to understand the effect of the study drug. You might experience less bleeding, so may receive fewer platelet transfusions and so avoid their risks.

**What are the possible disadvantages and risks of taking part?**
It is possible that those patients who do not receive the study drug (TXA) experience more signs and symptoms of bleeding and may need more platelet or blood transfusions than those who do, however whether this is the case is not yet known.

The study is continuously monitored by coordinators and an independent monitoring committee to ensure that if one treatment emerges as clearly better, or if new information becomes available, the study will stop.

**What are the side effects of any treatment received?**

The study drug (TXA) is used to treat bleeding associated with a number of other conditions and is already used in other settings where bleeding is a problem. Whilst in theory there is a slight risk that the study drug could cause unwanted blood clots, a thorough review of published studies has not shown this to be a problem in other conditions and there are no bad side effects in the short term. We don’t know for certain if this will be the same for people with your condition. Therefore, the study doctors, as well as your doctors and nurses, will monitor you carefully for blood clots as well as any other problems in order to give you the best available care if there are any problems.

What happens if I don’t want to carry on with the study?

If you change your mind about taking part, you just need to tell the research nurse or doctor that you don’t want to be in the study any more. You can do this at any time. Your ward doctors and nurses will carry on giving you all the treatments and care you need for your illness. If you withdraw from the study, we will keep any information we have collected up to that point.

Number of patients in the trial

We are planning to enrol 616 patients, from hospitals in the UK and Australia. This number of patients will give us results that are meaningful. With a smaller number it would be difficult to tell whether the TXA was helpful, or whether any difference found was just chance. We anticipate that it will take 4 years to enrol 616 patients.

**What if new information becomes available?**

Your study doctor will discuss with you any important new findings that may develop during the study and which might affect your willingness to participate.

Also, on receiving new information, your study doctor might consider it to be in your best interests to withdraw you from the study. He/she will explain the reasons why and arrange for your normal care to continue.

**What happens if something goes wrong?**

If by taking part in this study you suffer any harm due to the study itself, you have a right to complain or take legal action against NHSBT unless that harm results from a negligent act or omission by the hospital, in which case it is the hospital’s responsibility. Should the harm result from the contents of a blood transfusion you do of course have rights under the Consumer Protection Act. If you wish to complain or have any concerns about any aspect of the way you have been approached or treated during the course of this study the normal National Health Service complaints mechanisms are available to you. Please contact Patient Advisory Liaison Service (PALS) if you have any concerns about the care you have received or as an initial point of contact if you have a complaint. Please telephone XXXXX XXXXXX or e-mail insert relevant e-mail address, you can also visit PALS by asking at any hospital reception.

**Will my taking part in this study be kept confidential?**

It is a requirement that your involvement in this study be noted in your medical records. Direct access to your records will be required by members of the research team to check the information collected for the study. Research staff from *[insert hospital name]* will keep your name and contact details confidential and will not pass this information to NHS Blood and Transplant (NHSBT). Research staff from *[insert hospital name]* will use this information as needed; to contact you about the trial, make sure that relevant information about the study is recorded for your care, and to oversee the quality of the study. NHSBT will only receive information without any identifying information.

At all times your details will be handled only by fully trained staff and will remain confidential. All information which is collected about you during the course of the research will be anonymised, kept strictly confidential and will be stored securely in coded form. The people who analyse the information will not be able to identify you and will not be able to find out your name or contact details. If you consent to take part in the research the people conducting the study will abide by the Data Protection Act 2018, and the rights you have under that act. NHSBT is the sponsor for this trial and will act as the data controller. This means that NHSBT are responsible for looking after your information and using it properly. Certain individuals from NHSBT and regulatory organisations may look at your medical and research records to check the accuracy of the research study.

You can find out more about how we use your information by contacting [treatt@nhsbt.nhs.uk](mailto:treatt@nhsbt.nhs.uk)**.** NHSBT will keep non-identifiable information about you for 5 years after the study has finished.

Your rights to access, change or move your information are limited, as we need to manage your information in specific ways in order for the research to be reliable and accurate. To safeguard your rights, we will use the minimum personally-identifiable information possible.

**Informing your GP**

We will write a letter to your GP so that s/he knows you are in the trial and have been taking the study treatment (either TXA or placebo).

What will happen to the results of the research study?

It is likely that a report containing the results of this study will be written, presented at scientific meetings and published in scientific magazines following the end of the patient recruitment. Your identity will not be made known. The results of this trial will inform clinical decision making about the use of TXA in haematology patients. If you would like to know the results of this study when it is published, please let the research nurse know your contact details.

Who is organising and funding the research?

This study is being organised and funded by NHS Blood and Transplant (NHSBT).

Who has reviewed the study?

All research that involves NHS patients or staff, information from NHS medical records or uses NHS premises or facilities must be approved by a NHS Research Ethics Committee before it goes ahead. Approval does not guarantee that you will not come to any harm if you take part. However, approval means that the Committee is satisfied that your rights will be respected, that any risks have been reduced to a minimum and balanced against possible benefits, and that you have been given sufficient information on which to make an informed decision to take part or not.

This study has been reviewed and approved by the South-Central Oxford C Research Ethics Committee, which has approved this study at a national level, and by the local research department at your hospital.

Costs/Reimbursement

You will not incur any costs from participating in this study.

What if I need further information or have any concerns?

Now or during the course of the study, if you have any concerns or other questions about this study or the way it has been carried out, you should contact your local study doctor and team whose contact details you will have been given. Alternatively, you can contact one of the national study coordinators **at treatt@nhsbt.nhs.uk or by calling the trial manager on 01865 387903**. You will be given a copy of the information sheet and a signed consent form to keep.

**Thank you for taking the time to read through this information sheet and considering taking part in this study.**

***Insert Hospital header***


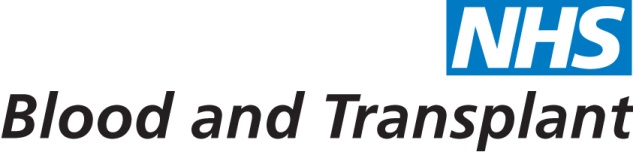


**PARTICIPANT INFORMATION SHEET**

**TREATT: TRial to EvaluAte Tranexamic acid therapy in Thrombocytopenia**

***Effectiveness of a supplementary drug treatment.***

REC reference: 14/SC/1290

We are inviting you to take part in a research study. Before you decide whether to take part it is important for you to understand why the study is being done and what it will involve. Please take time to read the following information carefully and discuss it with friends and relatives if you wish. Ask us if there is anything that is not clear or if you would like more information.

**Why have I been invited?**

Patients with haematological diseases often develop a very low platelet count. This may be either as a result of the disease itself or the treatment, including chemotherapy and stem cell transplantation. It is expected that during your hospital stay you will develop a low platelet count. Patients with low platelet counts sometimes experience bleeding. Usually this is minor, for example some bruising or red blotches on your skin, or maybe a short nose-bleed. A platelet transfusion is often given to raise the low platelet count. But we now know that despite platelet transfusions, many patients continue to experience some bleeding when platelet counts are low.

What is the purpose of the study?

This study is to find out if giving a drug called tranexamic acid (TXA) whilst platelet counts are low will reduce bleeding. We would give this drug to some patients in addition to platelet transfusions.

**Do I have to take part?**

No, it is up to you to decide whether or not to take part. This choice will not affect your current or future care; your doctors and nurses will still care for you and give you all the treatments you need. You would be free to come out of the study at any time and without giving a reason. This will not affect the standard of care you receive.

How does the study work?

We don’t know whether TXA as well as all the usual treatments will be better at reducing bleeding or not. The best way to find out is to see how people who are given it do, compared to people who are not. This means that patients taking part in the study will be divided into two groups. Patients in one group will receive the study drug (TXA); patients in the other group will receive a placebo. The two groups will be similar so that a fair comparison can be made. The groups will be selected at random using a computer, which needs no personal information about you. The study drug and the placebo look the same so you, the doctors and the ward nurses don’t know which group you are in.

What will happen to me if I take part?

If you agree to take part, you will first be asked to sign a consent form. The research nurse will register you in the trial and check your daily blood results to see what your platelet count is. When your platelet count is 50 (50 x 10^9^/L) or less the trial computer system will allocate you to a treatment (study drug or placebo). Neither the research nurse nor the ward nurses will know what treatment you are having. When your platelet count is 30 (30 x 10^9^/L) or less the ward nurses will start the study treatment. To begin with you will receive the study treatment through a drip, 3 times a day. You can change to tablets if you are well enough to swallow them. There will be 3 tablets 3 times a day. You will continue with all your normal treatment as well.

Your doctor will arrange for you to have a platelet transfusion if your platelet count is very low, below 10 (10 x 10^9^/L), or if you have any signs of bleeding.

A member of the research team will check your ward charts, medical notes and blood results on a daily basis to collect blood results that we need for this study.

**Fibrinolysis sub-study.**

We would also like to take some extra blood samples when your platelet count is low. These blood samples will be used to see if there are any changes in the way your blood clots during your treatment.

This sub-study is optional. You can choose to participate in the main study but not the fibrinolysis sub-study.

If you agree to this sub-study, we will take an extra blood sample (approximately three teaspoonful’s) when you initially agree to take part, or the following day, and then an extra blood sample (approximately three teaspoonful’s) three times a week (e.g. Monday, Wednesday and Friday) when your platelet count is low. During your treatment you will require regular blood tests, and the extra blood samples required for this study will be taken, if possible, at the same time as your routine samples. The blood samples will usually be taken via the “Hickman line” or “PICC line” that has been inserted as part of your treatment. However, very occasionally, it may be necessary to take the blood samples from a vein using a needle.

Participant’s responsibilities.

What do I have to do?

It is likely that you will be in hospital for some of your treatment. We would like to record signs and symptoms of bleeding when your platelet count is low. We know from experience that on many of these days you will not have any bleeding.

When you are on the ward, one of the research nurses helping with this study will come and see you each morning to ask you questions about signs and symptoms of bleeding and to look at your skin and in your mouth. This information will be recorded in a bleeding assessment form.

We would like to collect information about bleeding for 30 days after you start the study treatment. If you go home before the 30 days are finished, we will give you a simple diary card to fill in yourself with details of any signs or symptoms of bleeding.

If your platelet count is still low, less than 30, (*30 x 10^9^/L*), when you go home we will give you the study treatment to take as tablets by mouth 3 times a day until your platelet count is 30 or more or until you have been receiving the study treatment for 30 days.

We would like to ask you to complete 2 short questionnaires which have questions about your general wellbeing and quality of life. These would be completed on 2 occasions when you are in hospital. We would also like to contact you 1 month after starting the study treatment and again 3 months after that to see how you are. This contact could be either in the outpatient clinic or over the telephone. If you agree to having the additional blood tests taken you will have extra blood samples taken three times a week until your study treatment stops.

What are the possible benefits of taking part?

We do not know if being in this study will help you, but it will help doctors who treat haematology patients in the future to understand the effect of the study drug. You might experience less bleeding, so may receive fewer platelet transfusions and so avoid their risks.

What are the possible disadvantages and risks of taking part?

It is possible that those patients who do not receive the study drug (TXA) experience more signs and symptoms of bleeding and may need more platelet or blood transfusions than those who do, however whether this is the case is not yet known.

The study is continuously monitored by coordinators and an independent monitoring committee to ensure that if one treatment emerges as clearly better, or if new information becomes available, the study will stop.

**What are the side effects of any treatment received?**

The study drug (TXA) is used to treat bleeding associated with a number of other conditions and is already used in other settings where bleeding is a problem. Whilst in theory there is a slight risk that the study drug could cause unwanted blood clots, a thorough review of published studies has not shown this to be a problem in other conditions and there are no bad side effects in the short term. We don’t know for certain if this will be the same for people with your condition. Therefore, the study doctors, as well as your doctors and nurses, will monitor you carefully for blood clots as well as any other problems in order to give you the best available care if there are any problems.

What happens if I don’t want to carry on with the study?

If you change your mind about taking part, you just need to tell the research nurse or doctor that you don’t want to be in the study any more. You can do this at any time. Your ward doctors and nurses will carry on giving you all the treatments and care you need for your illness. If you withdraw from the study, we will keep any information we have collected up to that point.

Number of patients in the trial

We are planning to enrol 616 patients, from hospitals in the UK and Australia. This number of patients will give us results that are meaningful. With a smaller number it would be difficult to tell whether the TXA was helpful, or whether any difference found was just chance. We anticipate that it will take 4 years to enrol 616 patients.

**What if new information becomes available?**

Your study doctor will discuss with you any important new findings that may develop during the study and which might affect your willingness to participate.

Also, on receiving new information, your study doctor might consider it to be in your best interests to withdraw you from the study. He/she will explain the reasons why and arrange for your normal care to continue.

**What happens if something goes wrong?**

If by taking part in this study you suffer any harm due to the study itself, you have a right to complain or take legal action against NHSBT unless that harm results from a negligent act or omission by the hospital, in which case it is the hospital’s responsibility. Should the harm result from the contents of a blood transfusion you do of course have rights under the Consumer Protection Act. If you wish to complain or have any concerns about any aspect of the way you have been approached or treated during the course of this study the normal National Health Service complaints mechanisms are available to you. Please contact Patient Advisory Liaison Service (PALS) if you have any concerns about the care you have received or as an initial point of contact if you have a complaint. Please telephone XXXXX XXXXXX or e-mail *insert relevant e-mail address*, you can also visit PALS by asking at any hospital reception.

**Will my taking part in this study be kept confidential?**

It is a requirement that your involvement in this study be noted in your medical records. Direct access to your records will be required by members of the research team to check the information collected for the study*.* Research staff from [*insert hospital name]* will keep your name and contact details confidential and will not pass this information to NHS Blood and Transplant (NHSBT). Research staff from *[insert hospital name]* will use this information as needed; to contact you about the trial, make sure that relevant information about the study is recorded for your care, and to oversee the quality of the study. NHSBT will only receive information without any identifying information.

At all times your details will be handled only by fully trained staff and will remain confidential. All information which is collected about you during the course of the research will be anonymised, kept strictly confidential and will be stored securely in coded form. The people who analyse the information will not be able to identify you and will not be able to find out your name or contact details. If you consent to take part in the research the people conducting the study will abide by the Data Protection Act 2018, and the rights you have under that act. NHSBT is the sponsor for this trial and will act as the data controller. This means that NHSBT are responsible for looking after your information and using it properly. Certain individuals from NHSBT and regulatory organisations may look at your medical and research records to check the accuracy of the research study.

You can find out more about how we use your information by contacting treatt@nhsbt.nhs.uk. NHSBT will keep non-identifiable information about you for 5 years after the study has finished.

Your rights to access, change or move your information are limited, as we need to manage your information in specific ways in order for the research to be reliable and accurate. To safeguard your rights, we will use the minimum personally-identifiable information possible.

**Informing your GP**

We will write a letter to your GP so that s/he knows you are in the trial and have been taking the study treatment (either TXA or placebo).

What will happen to the results of the research study?

It is likely that a report containing the results of this study will be written, presented at scientific meetings and published in scientific magazines following the end of the patient recruitment. Your identity will not be made known. The results of this trial will inform clinical decision making about the use of TXA in haematology patients. If you would like to know the results of this study when it is published, please let the research nurse know your contact details.

Who is organising and funding the research?

This study is being organised and funded by NHS Blood and Transplant.

Who has reviewed the study?

All research that involves NHS patients or staff, information from NHS medical records or uses NHS premises or facilities must be approved by a NHS Research Ethics Committee before it goes ahead. Approval does not guarantee that you will not come to any harm if you take part. However, approval means that the Committee is satisfied that your rights will be respected, that any risks have been reduced to a minimum and balanced against possible benefits, and that you have been given sufficient information on which to make an informed decision to take part or not.

This study has been reviewed and approved by the South-Central Oxford C Research Ethics Committee, which has approved this study at a national level, and by the local research department at your hospital.

Costs/Reimbursement

You will not incur any costs from participating in this study.

What if I need further information or have any concerns?

Now or during the course of the study, if you have any concerns or other questions about this study or the way it has been carried out, you should contact your local study doctor and team whose contact details you will have been given. Alternatively, you can contact one of the national study coordinators **at treatt@nhsbt.nhs.uk or by calling the trial manager on 01865 387903** You will be given a copy of the information sheet and a signed consent form to keep.

**Thank you for taking the time to read through this information sheet and considering taking part in this study.**
